# *Lactobacillus* Cell Surface Proteins Involved in Interaction with Mucus and Extracellular Matrix Components

**DOI:** 10.1007/s00284-020-02243-5

**Published:** 2020-10-20

**Authors:** Lidia Muscariello, Barbara De Siena, Rosangela Marasco

**Affiliations:** Dipartimento Di Scienze E Tecnologie Ambientali, Biologiche E Farmaceutiche, Università Degli Studi Della Campania “L. Vanvitelli”, via Vivaldi 43, 81100 Caserta, Italy

## Abstract

The gut microbiota is a complex microbial ecosystem where bacteria, through mutual interactions, cooperate in maintaining of wellbeing and health. Lactobacilli are among the most important constituents of human and animal intestinal microbiota and include many probiotic strains. Their presence ensures protection from invasion of pathogens, as well as stimulation of the immune system and protection of the intestinal flora, often exerted through the ability to interact with mucus and extracellular matrix components. The main factors responsible for mediating adhesion of pathogens and commensals to the gut are cell surface proteins that recognize host targets, as mucus layer and extracellular matrix proteins. In the last years, several adhesins have been reported to be involved in lactobacilli–host interaction often miming the same mechanism used by pathogens.

## Introduction

The gut microbiota is a complex microbial ecosystem where bacteria, through mutual interactions, cooperate in maintaining of wellbeing and health of the host. Indeed, intestinal bacteria play a key role in modulating immune system, metabolic pathways and in providing protection against invasion by pathogens. Intestinal microflora consists of about 10^13^–10^14^ organisms, with more than 1000 different species. Its alteration, called disbiosis, may cause damage to the host health. An important contribution to the activity of the intestinal microbiota is given by lactobacilli. They are among the most numerous bacteria in the proximal small intestine of healthy individuals where they adhere to the epithelium and the mucosal layers, contributing to the balance of the microbial ecosystem. Their presence provides protection from invasion of pathogens and stimulation of the immune response. Ability of *Lactobacillus acidophilus* S-layer protein A (SlpA) to inhibit bacterial infection by blocking cellular receptor DC-SIGN and murein hydrolase activity is a clear example of these features [1–3]. Moreover it has been also shown that the SlpA/DC-SIGN interaction plays a key role in the regulation of dentritic cells and T cells functions [1]. In light of all this, lactobacilli are increasingly used for their nutraceutic (nutribiotics) and pharmaceutic (pharmabiotics) properties. Particularly, pharmabiotics may be potential tools for the prophylaxis or treatment of enteric infections [4]. Adhesion ability of probiotic bacteria might contribute to their beneficial effects by favoring colonization and extending persistence in the gut. In adhesion processes of lactobacilli, surface proteins, so called adhesins, play a key role by interacting with host receptors. They are mainly multi-functional cytoplasmatic proteins, exerting moonlighting functions when expressed on cell surface as cell wall-anchored proteins. It has been reported that some of these proteins are glycosylated [5]. This review focuses on adhesins of different species of the genus *Lactobacillus* responsible for mediating adhesion to mucus layer and extracellular matrix proteins. These studies shed light on mechanisms through which lactobacilli exert their beneficial effects on human health.

### Mucus Binding Proteins

**Fig. 1:**
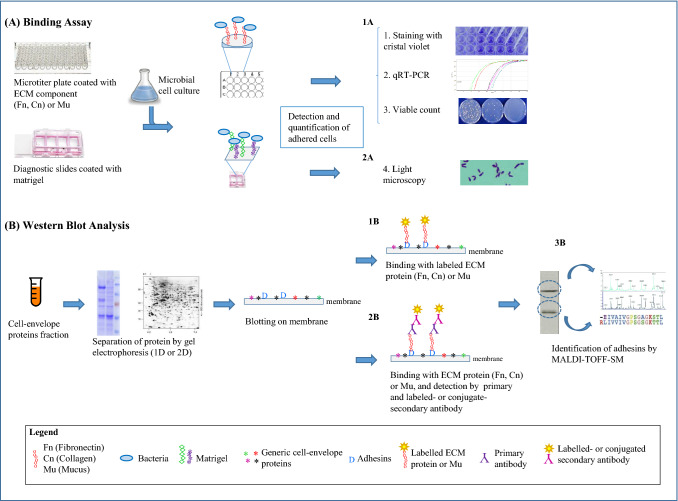
Graphic representation of the most common in vitro model systems described to study bacteria/host interaction. **a** Detection of bacterial adhesion to mucus (Mu) or ECM components, e.g. fibronectin (Fn) and collagen (Cn). Binding assay can be performed on microtiter plate, coated with one ECM component or mucus (upper), or on diagnostic slides coated with matrigel (lower), which contains mostly Cn and laminin. Microbial cell culture of the strain under study is added in each well and, after washing, adhered cells can be detected and quantified by different methods: **1a**—staining with crystal violet [61], qRT-PCR [77] or viable count [49], when microtiter plate is used; **2a**—by light microscopy, when diagnostic slides are used [73]. **b** Identification of proteins involved in the bacteria/host interaction. Extracted surface proteins are separated by mono-dimensional (1D) or two-dimensional (2D) gel-electrophoresis and western blotted by using labeled ECM or mucus components [9] (**1b**), or specific polyclonal antibodies and labelled or conjugate secondary antibody [77] (**2b**). Identification of putative adhesins may be obtained by MALDI-TOF Mass Spectrometry (**3b**)

Studies on lactobacilli/host interaction have been performed almost exclusively on in vitro model systems (Fig. 1) [6]. Adhesion abilities of lactobacilli to the mucosa have been particularly documented [7, 8]. Mucus is a highly dynamic matrix coating the epithelial cells and protecting the host against colonization by pathogens. In the colon, mucus matrix is made up of a compact inner layer that is largely sterile and an outer layer composed of mucus, intestinal bacteria and dietary material. The main structural components of mucus layer are mucins, a family of high molecular weight, heavily glycosylated proteins. Important characteristics of mucins are their abilities to function as lubricants and chemical barriers. Mucus provides a habitat for commensal bacteria, which are necessary for eliciting or modulating the host immune system; however, it is also considered to be critical for bacterial adhesion to the gut. To allow adhesion to different mucin glycans, lactobacilli have evolved the ability to express several adhesins including moonlighting proteins (Table 1). In *Lactobacillus reuteri*, many mucus binding proteins have been identified. Indeed, *L. reuteri* 104R MapA was among the first adhesins to be described [9]. This protein is homologue to the collagen binding protein CnBP of *Lactobacillus crispatus* and is also able to adhere to collagen and Caco-2 cells [10]. A MapA degradation product showed antimicrobial activity, suggesting pleiotropic functions for MapA [11]. Furthermore, Matsuo et al. [12] showed that MapA binds to the ANXA13 and PALM proteins on the Caco-2 cell membrane. Roos and Jonsson [13] described an extracellular mucus binding protein (MUB) in *L. reuteri* ATCC 53608 (strain 1063, isolated from pig) whose crystal structure suggested an immunoglobulin binding activity [14]. This protein belongs to a family of structurally similar cell surface proteins that contains an N-terminal secretion signal peptide, a C-terminal LPxTG motif, followed by a C-terminal helix and a positively charged tail. The LPxTG motif allows binding of MUB to peptidoglycan after cleavage by the sortase SrtA. By flow-cytometry, it has been also shown that MUB is involved in bacterial auto-aggregation mechanisms [15]. The presence in MUB of two different types of repeats for binding to mucus and mucin, named Mub1 and Mub2, has also been reported. Each repeat has a mucin binding domain and an immunoglobulin binding domain. Structural and functional analyses of MUB repeats have shown that they recognize terminal sialic acid residues both in mucin chains and immunoglobulins (Table 2) [8]. Using atomic force microscopy, Gunning et al. [16] suggested a multiple binding model of MUB to mucin chains that requires a MUB self-interaction mediated by its modular structure. That would explain the considerable strength of the MUB/mucin binding. Another example of *L. reuteri* mucus binding protein with LPxTG motif is the CmbA protein, which mediates binding of *L. reuteri* ATCC PTA 6475 to Caco-2 cells and mucus [17]. Indeed, a mutant strain with a deletion in *cmbA* was unable to adhere to mucus, suggesting that CmbA is of primary importance for the adhesive properties of this strain. In *L. reuteri* JCM112 the CmbA homologue is the Lar_0958 protein, containing six repeat domains. One of these shows structural homology with the Ig-like inter-repeat domain of *Listeria monocytogenes* internalins [18]. Recently, it has been reported that CmbA and MUB exert immunomodulatory properties in the gut through the Th1 promoting interaction with C-type lectins on human monocyte-derived dendritic cells [19]. The diversity and variability in abundance of *L. reuteri* MUBs reflect the different mucus binding ability of several strains [15]. Adhesins involved in mucin binding have been also reported in other lactobacilli as *Lactobacillus fermentum, Lactobacillus acidophilus, Lactobacillus johnsonii* and *Lactobacillus plantarum.* It has been demonstrated that *L. fermentum* BCS87 32-Mmubp, a component of an ABC transporter system, is a mucus and mucin binding protein, suggesting that membrane transport proteins may have more than one function [20]. Chatterjee et al. [21] have shown that a 93-amino acid mucin binding domain (MBD93) of the LAF_0673 protein from *L. fermentum* IFO 3956 is sufficient for mucin binding and protection from enteric pathogens invasion (Table 2). Recently, immunomodulatory activity of MUB from *L. acidophilus* has been reported. It involves the Toll-like receptor 4 signaling pathway and causes the activation of mitogen-activated protein kinase signaling pathway (MAPK) [22]. Along with *L. reuteri, L. plantarum* is among the lactobacilli most studied for its adhesion ability. Numerous studies have characterized interactions between some strains of this bacterium and mucus. The first mucin binding protein to be identified in *L. plantarum* WCFS1 was the lectin-like mannose specific adhesin (Msa) [23]. No correlation was found between type I and II MUB domains of Msa identified in different strains and mannose adhesion ability [24]. Holst et al. [25] showed that the diversity in mannose binding ability among *L. plantarum* strains is related to variations of *msa* expression levels. The *L. plantarum* probiotic strain Lp9 was found to possess genes for four MUB proteins [26], including *lp_1643* that encodes a protein with six tandem MUB domains; the last two domains (Mubs5s6) were functional to the binding with different gut mucosa components and reduced the binding of enterotoxigenic *Escherichia coli* cells to the enterocytes (Table 2). In addition, Mubs5s6 showed affinity for calcium and glucose, which were supposed to mediate pathogen adhesion to host cells [27]. It has been reported that the flagella protein FliC of several pathogens is involved in binding to mucin, confirming the role of flagella in adhesion processes [28–30]. Interestingly, the FliC predicted domain was also located in lp_2486, lp_1643 and lp_2486 orthologues of some *L. plantarum* infant isolated [31]. The authors suggest that these strains could be considered potential probiotics, capable of providing protection against the invading pathogens.

**Table 1:** Adhesins in different species of *Lactobacilli* and their multiple functions

*Lactobacillus* specie/strain	Cell surface protein	Adesion targets/functions	References
Mucus binding proteins			
* Lactobacillus reuteri* 104R	MapA	• Mucus, collagen and Caco-2 cells	[9, 10, 12]
* Lactobacillus reuteri* ATCC 53608	MUB	• Mucus, mucin and immunoglobulin • Involvement in bacterial auto-aggregation • Immunomodulatory activity	[8, 14–16, 19]
* Lactobacillus reuteri* ATCC PTA6475	CmbA	• Mucus and Caco-2 cells • Immunomodulatory activity	[13, 17, 19]
* Lactobacillus fermentum* BCS87	32-Mmubp	• Mucus and mucin • Component of an ABC transporter system	[20]
* Lactobacillus fermentum* IFO 3956	LAF_0673	• Mucin • Protection from enteric phatogens invasion	[21]
* Lactobacillus acidophilus*	MUB	• Mucus • Immunomodulatory activitiy	[22]
* Lactobacillus plantarum* WCFS1	Msa	• Mannose residues present on the intestinal cells	[23–25]
* Lactobacillus plantarum* Lp9	lp_1643	• Mucus adhesion • Inhibition of enterotoxigenic *Escherichia coli* binding to enterocytes	[26, 27]
Collagen/fibronectin binding proteins			
* Lactobacillus reuteri* NCIB 11951	Cnb	• Collagen	[40]
* Lactobacillus crispatus* JCM 5810	CbsA	• Collagen • Involvement in bacterial auto-aggregation • Immunomodulatory activity	[50, 51]
* Lactobacillus crispatus* (K2-4–3 and K313 strains)	SlpB	• Collagen	[52, 53]
* Lactobacillus casei* BL23	LCABL_01820	• Collagen • Fibronectin	[55]
	FbpA	• Fibronectin	[69]
* Lactobacillus plantarum* 91	Cbp	• Collagen • Inibition of *Escherichia coli* O157:H7 binding to collagen	[56]
* Lactobacillus fermentum* 3872	CBP	• Collagen • Inhibition of *Campylobacter jejuni* binding to collagen	[50, 51]
* Lactobacillus acidophilus* NCFM	FbpA	• Fibronectin and Caco-2 cell	[58]
	FbpB	• Mucin and fibronectin	[59]
Moonlighting binding proteins			
* Lactobacillus johnsonii* NCC533	EF-Tu	• Mucin and Caco-2 cell • Immunomodulatory activity • Protein synthesis elongation factor	[63]
	GroEL	• Mucin and intestinal epitelial cell • Immunomodulatory activity • Induction of *Elicobacter pylori* aggregates • Molecular chaperone	[72]
* Lactobacillus reuteri* JCM1081	EF-Tu	• Mucin • Protein synthesis elongation factor	[66]
* Lactobacillus crispatus* ST1	Eno (enolase), GS (glutamine synthetase), GPI (glucose-6-phosphate isomerase)	• Collagen • Central Carbon metabolism enzymes	[73, 78]
* Lactobacillus plantarum* 299v	Eno, GAPDH (glyceraldehyde-3-phosphate dehydrogenase)	• Fibronectin • Glycolytic enzymes	[76]
* Lactobacillus plantarum* LM3	EnoA1 (enolase A1)	• Fibronectin, collagen and Caco-2 adhesion • Immunomodulatory properties • Biofilm development • Glycolytic enzyme	[77, 79, 80]
	PDHB (E1 beta-subunit of pyruvate dehydrogenase)	• Fibronectin, collagen • Biofilm development • Glycolytic enzyme	[81, 82]
* Lactobacillus acidophilus* NCFM	Elongation factor G	• Mucin • Elongation factor in protein synthesis	[90]
	Pyruvate kinase	• Mucin • Glycolytic enzyme

**Table 2:** Adhesion mechanisms of some surface cell proteins to host targets

Adhesin	Binding domain	Host target	References
MUB (3269 aa) (*Lactobacillus reuteri* ATCC 53608)	Mub type 1 and type2 repeats	Terminal sialylated mucin glycans	[8, 16]
LAF_0673 (1059 aa) *Lactobacillus fermentum* IFO 3956	MBD93, 93 aa residues (890–982) at the C-terminal, with Ser57, Pro58, Ile60, Tyr63 and Ala65 residues likely involved in binding	Mucin glycans (N-acetylgalactosamine, N-acetylglucosamine, galactose, and sialic acid)	[21]
Lp_1643 (2219 aa) *Lactobacillus plantarum* Lp9	Mubs5s6, 1198 aa fragment at the C-terminal, with two mucus binding domains	Mice intestinal mucus, pig gastric mucus, HT-29 and Caco-2 cell lines, and surface components of human enteric tissues (cytokeratins, Hsp90 and laminin)	[27]
SlpB (440 aa) *Lactobacillus crispatus* K313	379 aa residues (1–379) at the N-terminal	Type I and IV collagen	[46]
EnoA1 (442 aa) *Lactobacillus plantarum* LM3	67 aa residues (73–140) at the N.terminal	Type I collagen	[79]

### Fibronectin and Collagen Binding Proteins

The extracellular matrix (ECM) is an important constituent of animal tissues, whose composition and structure differs from one tissue to another. Being ubiquitously and profusely distributed, some of its components, such as collagen and fibronectin, can be adhesion targets for bacterial pathogens as well as for commensal bacteria [32, 33]. Indeed, bacteria express several cell surface proteins that specifically interact with ECM, among which the most studied are the proteins called MSCRAMM (microbial surface components recognizing adhesive matrix molecules). Pathogens and commensals often share the same type of adhesins in colonization processes. Therefore, many studies investigated the role of adhesins expressed on cell surface of commensal bacteria, as anti-adhesion agents for the prevention of infections. Collagen is the major glycoprotein of connective tissues that forms aggregates stabilized by triple helical domain interactions. Collagens are involved in many important functions like providing the scaffold for the attachment of other ECM components [34]. Among different types of collagens described so far, collagens I e V are the most commonly encountered and are the main targets of pathogens for adhesion to host tissues [35–39]. Wide diversity in the collagen adhesion properties has been also recorded among the different probiotic species of lactobacilli (Table 1). One of the first collagen I binding proteins to be described in a probiotic strain was Cnb of *L. reuteri* NCIB 11951 [40]. A well characterized example of collagen I and IV-targeting adhesin is *L. crispatus* JCM 5810 CbsA, a component of the proteinaceous surface layer involved in bacterial aggregation and adhesion as well as in immunomodulation processes [41–43]. The N-terminal two-thirds of CbsA bind to collagen while the C-terminal region anchors the protein to the cell wall through binding to lipoteichoic and teichoic acids [44]. Its cell wall binding domain has a high similarity with the SlpB C-terminal region (LcsB), another S-layer collagen binding protein identified in *L. crispatus* K2-4-3 and able to bind to collagen via a N-terminal domain. It has been proposed that the LcsB region may be sufficient to target heterologous proteins to the probiotic bacteria cell surface [45]. Moreover, by using truncated recombinant SlpB proteins from *L. crispatus* K313, Sun et al. [46] mapped the cell wall binding region and the collagen I and IV binding domain in the C and N-terminal regions of the protein, respectively (Table 2). Additionally, *L. crispatus* SlpB was also reported to enhance the antimicrobial activity of nisin [47]. By the shotgun phage-display technique, that provides the identification of host receptor interacting peptides within a protein sequence, Munoz-Provencio and Monedero [48] identified the product of the *L. casei* LCABL_01820 gene as a protein able to bind to collagen and fibronectin. A surface layer collagen binding protein (Cbp), with a counteracting activity versus *E. coli* O157:H7 binding, has been also identified in *L. plantarum* 91 [49]. Recent studies have reported the ability of both *L. fermentum* 3872 whole cells and its putative collagen binding protein (CBP) to inhibit binding of *Campylobacter jejuni* to collagen I [50]. In previous works, the *cbp* gene was reported to code a protein consisting of an N-terminal A domain for collagen adhesion followed by multiple repeats of B domains and a C-terminal LPxGT domain necessary for cell wall anchoring [51]; B domains form stalks required for a correct surface localization of the A region. Genomic analysis of *L. fermentum* 3872 showed that full and partial copies of the *cbp* gene were localized on a plasmid and on the chromosome, respectively [50, 51].

Fibronectin is a large dimeric multi-domain glycoprotein whose monomers are linked covalently by two C-terminal disulphide bonds. It is found in body fluids and in the ECM of different connective tissues including intestinal epithelia. Each monomer consists of three types of units variously repeated, responsible for interaction with other ECM components and integrins. In addition to playing an important role in cell adhesion, growth, migration and differentiation, fibronectin is also a common target for bacterial adhesins of either pathogens or commensals [52, 53]. Fibronectin binding proteins (FnBps) have been mainly characterized in pathogens, where they can also act as virulence factors. The majority of these belong to the MSCRAMM protein family and present additional actions to the simple adhesion activity [53, 54]. Many evidence show that FnBps can change physiological functions of fibronectin thus contributing to development of infectious disease [55]. Few FnBps have been identified and characterized in probiotics, and many of them are analogous to those identified in pathogens, although they were shown to lack pathogenic functions (Table 1) [56, 57]. FbpA, a homolog of FnBps found in pathogens, was identified in *L. acidophilus* NCFM. This protein contains a fibronectin binding domain similar to that of *Staphylococcus aureus* Fbp54 [58]. A *fbp*A mutant showed a decrement in adhesion to Caco-2 cells, suggesting that bacterial adhesion to intestinal cells is achieved through interactions of multiple factors. More recently, in *L. acidophilus* NCFM a second fibronectin binding protein (FbpB) was identified as an S-layer associated protein, which is also involved in adhesion with mucin [59]. A fibronectin type III domain has been identified at the FbpB C-terminal end. To further characterize the FbpB–fibronectin interaction, a recent report described the heterologous expression of a pure and biologically active form of the *L. acidophilus* FbpB [60]. The goal of this type of study was to identify new drug delivery strategies in the gut for therapeutic purpose. A surface exposed FbpA-homologue protein was also characterized in *L. casei* BL23 [61]. This protein lacks signals for secretion and membrane anchoring and is also present in the cytosol. Such evidences suggest that FbpA of *L. casei* may exert moonlighting functions.

## Role of Moonlighting Proteins in Adhesion of Lactobacilli

In lactobacilli some of the adhesion factors described so far are cytoplasmatic multi-functional proteins that exert moonlighting functions when expressed on cell surface. No signal peptide responsible for secretion or hydrophobic membrane-spanning regions has been identified in their sequence, so it is not known how they are placed on the cell surface. The term moonlighting was introduced to indicate proteins able to perform two or more physiologically important functions. Moonlighting proteins have been detected in plants, animals, yeast and bacteria, where they are involved in biologically relevant processes. Today we know that more than 100 cytoplasmatic proteins, mainly metabolic enzymes and molecular chaperones, are moonlighting proteins with activity of adhesion or modulation of cell signaling processes. Some of these are secreted soluble proteins often with function of immune system modulation. Many moonlighting proteins have been described in pathogenic microorganisms where they often play a key role in infection or virulence [62]. Despite their important role, few papers focused on moonlighting proteins of probiotic bacteria. It has been reported that several species of lactobacilli expose at their surface moonlighting proteins that can compete with pathogens for the same host receptors in the human gut (Table 1). The first surface moonlighting protein to be identified in lactobacilli was the *L. johnsonii* NCC533 EF-Tu protein synthesis elongation factor. EF-Tu recombinant protein was able to bind to mucin in a pH dependent manner and to induce a proinflammatory response [63]. The EF-Tu elongation factor was also reported to contribute to the *L. plantarum* 423 adhesion to Caco-2 cells and to have up-regulated expression in *L. acidophilus* ATCC 4356 cells when exposed to stressful conditions in the gut [64, 65]. More recent studies found that the EF-Tu moonlighting protein of *L. reuteri* JCM1081 shows a pH dependent binding to mucin that involves sulphate carbohydrates but not sialic acid [66]. GroEL heat shock proteins have been found at the cell surface of several mucosal pathogens where they mediate cell attachment and immune modulation [67–71]. Bergonzelli et al. [72] described the ability of the *L. johnsonii* NCC533 GroEL to bind to mucin and intestinal epithelial cells, to aggregate *Helicobacter pylori* cells and to stimulate IL-8 release in macrophages and HT-29 cells. As shown also for many pathogens, some surface glycolytic enzymes such as glyceraldehyde 3-phosphate dehydrogenase (GAPDH) and enolase have been found to act as adhesins in lactobacilli [62]. In *L. crispatus* ST1 glutamine synthetase (GS) and glucose-6-phosphate isomerase (GPI), along with enolase and GAPDH, are proteins associated to cell surface at acid pH and released at pH 8 and in the presence of the human antimicrobial peptide LL-37. Furthermore, binding of purified GS and GPI to type I collagen occurs stronger at acid pH [73]. According to data previously reported for the *L. plantarum* GAPDH [74], Kaiulainen et al. [73] suggested that incorporation into *L. crispatus* cell wall of these proteins is affected by changes in cellular permeability. More recently it was also demonstrated that *L. crispatus* enolase and GS have a role in protecting epithelium against *Neisseria gonorrhoeae* infections [75]. *L. plantarum* 299V GAPDH and enolase were also found to mediate adhesion to fibronectin whereas only GAPDH was able to weakly bind to mucin [76]. By immune electron microscopy, the surface localization of the *L. plantarum* LM3 alfa-enolase (EnoA1) was demonstrated, along with its ability to bind fibronectin [77]. The presence of two expressed *eno* genes in this strain (*enoA1* and *enoA2*), allowed isolation of the mutant strain LM3-CC1 (Δ*enoA1*), whose reduced fibronectin binding ability demonstrated, unequivocally, the involvement of EnoA1 in binding to fibronectin [77]. As previously reported for *L. crispatus* enolase [78], the *L. plantarum* EnoA1 can also adhere to collagen I and by an in vitro deletion analysis a fragment spanning from the 73^rd^ to the 140^th^ amino acid residues was shown to be sufficient for binding [79]. By means of comparative analysis between LM3 and its isogenic LM3-CC1 mutant, it has been demonstrated that EnoA1 is involved in immunostimulation of Caco-2 cells and in biofilm development [80]. Moreover, the ability of the *L. plantarum* E1 beta-subunit of pyruvate dehydrogenase (PDHB) to bind to fibronectin and collagen I was as well assessed [81, 82]. These studies described for the first time PDHB as an adhesin in a probiotic strain, being previously described as a fibronectin and plasminogen binding adhesin only in the pathogen *Mycoplasma pneumoniae* [83–85]. Similarly to EnoA1 and to other adhesins expressed on cell surface of different commensal or pathogen bacteria, *L. plantarum* PDHB is also involved in biofilm development [82]. The identification of lactobacilli adhesins involved in biofilm development, such as EnoA1 and PDHB, is of interest for the possible impact that the biofilm itself may have on persistence of the microorganism in the colon [86]*.* Furthermore, few studies showed that factors secreted from lactobacilli biofilms possess immunomodulatory properties [87, 88]. Recently, through comparative proteome analysis, some studies have correlated the expression of factors leading to probiotic characteristics of *L. acidophilus* NCFM with the presence in the growth medium of prebiotic carbohydrates or plant polyphenols. Growth on cellobiose, polydextrose or raffinose or in the presence of resveratrol or ferulic acid, stimulated adhesion of *L. acidophilus* to mucin. Under these growth conditions, changes in relative amount of known moonlighting proteins such as elongation factor G, GAPDH, pyruvate kinase and of other surface proteins were observed [89–91]. Furthermore the authors showed that recombinant *L. acidophilus* elongation factor G and pyruvate kinase significantly competed for adhesion of this bacterium to mucin. These results suggest the importance of the diet in modulating lactobacilli adhesive abilities and offer strategies for formulation of potential symbiotics. Other surface proteome studies showed the occurrence of higher amount of the phosphoglycerate mutase, glucosamine-6-phosphate deaminase, transcription elongation factor GreA and a small heat shock protein in the highly adhesive *Lactobacillus pentosus* CF1-43 N as compared to poorly adhesive strains. These data correlate the expression of some moonlighting proteins of a given strain with its probiotic properties and suggest their possible role as biomarkers for adhesion ability of *L. pentosus* strains [92].

## Conclusions

As reported in this review, it is evident that several surface layer proteins of lactobacilli have functions of adhesion to host epithelia and extracellular matrix components and have a role in modulation of the host immune response. These characteristics are assessed as strain-specific abilities and confer health benefit to the host. This overview highlights also the roles of lactobacilli moonlighting proteins in adhesion processes. Moreover, due to the fact that pathogens and probiotics often share similar mechanisms of adhesion [93], definition of binding domains within lactobacilli adhesins may contribute to the development of innovative antimicrobial therapies versus pathogens. Despite numerous studies conducted on moonlighting proteins, mechanisms by which they are secreted and bound to the bacterial cell surface remain to be elucidated. In this regard, understanding these processes in probiotics as well as in pathogens may be useful for the development of new therapeutic strategies and for the selection of new probiotic strains with enhanced beneficial effects on human health. Finally, the data reported in this review suggest that adhesins of lactobacilli, including moonlighting proteins, could play an important role in gut homeostasis. This reinforces the idea that lactobacilli, with their adhesins to be used as carriers for conveying antigens on intestinal surface, can be good candidates for development of live vaccines.
